# Co-expression of IL-15 enhances anti-neuroblastoma effectivity of a tyrosine hydroxylase-directed DNA vaccination in mice

**DOI:** 10.1371/journal.pone.0207320

**Published:** 2018-11-19

**Authors:** Madlen Marx, Maxi Zumpe, Sascha Troschke-Meurer, Diana Shah, Holger N. Lode, Nikolai Siebert

**Affiliations:** Department of Pediatric Hematology and Oncology, University Medicine Greifswald, Greifswald, Germany; University of Pécs Medical School, HUNGARY

## Abstract

Long-term survival of high-risk neuroblastoma (NB) patients still remains under 50%. Here, we report the generation, *in vitro* characterization and anti-tumor effectivity of a new bicistronic xenogenic DNA vaccine encoding tyrosine hydroxylase (TH) that is highly expressed in NB tumors, and the immune stimulating cytokine interleukin 15 (IL-15) that induces cytotoxic but not regulatory T cells. The DNA sequences of TH linked to ubiquitin and of IL-15 were integrated into the bicistronic expression vector pIRES. Successful production and bioactivity of the vaccine-derived IL-15- and TH protein were shown by ELISA, bioactivity assay and western blot analysis. Further, DNA vaccine-driven gene transfer to the antigen presenting cells of Peyer’s patches using attenuated *Salmonella typhimurium* that served as oral delivery system was shown by immunofluorescence analysis. The anti-tumor effect of the generated vaccine was evaluated in a syngeneic mouse model (A/J mice, n = 12) after immunization with *S*. *typhimurium* (3× prior and 3× after tumor implantation). Importantly, TH-/IL-15-based DNA vaccination resulted in an enhanced tumor remission in 45.5% of mice compared to controls (TH (16.7%), IL-15 (0%)) and reduced spontaneous metastasis (30.0%) compared to controls (TH (63.6%), IL-15 (70.0%)). Interestingly, similar levels of tumor infiltrating CD8^+^ T cells were observed among all experimental groups. Finally, co-expression of IL-15 did not result in elevated regulatory T cell levels in tumor environment measured by flow cytometry. In conclusion, co-expression of the stimulatory cytokine IL-15 enhanced the NB-specific anti-tumor effectivity of a TH-directed vaccination in mice and may provide a novel immunological approach for NB patients.

## Introduction

Neuroblastoma (NB) is a challenging malignancy originating from progenitor cells of the sympathetic nervous system and accounting for 10–15% of all childhood cancer deaths [1–3]. Although multimodal therapies, including passive Disialoganglioside (GD_2_)-directed immunotherapies, have significantly improved outcome of high-risk NB patients [4, 5], the 5-year event-free survival (EFS) rate still remains less than 50% [3]. This emphasizes the need for more effective therapeutic strategies that induce a long-lasting immune response to offer lifelong protection against cancer. To address this problem, a variety of vaccination strategies for active immunization of cancer patients [6–10] is under investigation and has shown promising results. Here, immunization with DNA vaccines which induce tumor-specific immune responses based on the activation of effector T cells offers several advantages compared to other immunization techniques. Not only is the design and production of DNA-based vaccines simple and cost-efficient, the DNA platform is also safe, stable, and well tolerated [11].

Since most malignancies arise from normal tissues and only express endogenous tumor-associated antigens (TAAs), which are associated with central or peripheral tolerance [12, 13], one of the major challenges of active immunotherapeutic strategies is to overcome such tolerance in order to induce specific anti-tumor immunity without adverse autoimmunity [14]. In this regard, the non-replicating *Salmonella typhimurium* strain SL7207, used as a vaccine delivery system, has been reported to be effective in inducing DNA vaccine-based immune response [15]. Other possibilities to increase the efficacy of DNA vaccination are integration of the ubiquitin sequence upstream of the TAA into the DNA plasmid allowing increased presentation of the TAA by MHC-I [16–18] and/or co-expression of genes encoding stimulatory molecules (e.g. chemokines or cytokines) [19]. Immunization with xenogeneic DNA vaccines, encoding highly homologous proteins of a foreign species, has been also shown to improve immunogenicity of DNA vaccination [20–22].

One of the well-known NB-specific TAA which serves as a clinical marker for diagnosis and follow-up of NB patients is the enzyme tyrosine hydroxylase (TH) [23, 24]. Previously, we reported the successful induction of a NB-specific immune response after TH-based DNA vaccination [16, 25, 26], showing an increased number of cytotoxic T lymphocytes (CTLs) in tumor but not in healthy TH-expressing tissues. Therefore, TH-based DNA vaccination may be a suitable therapeutic tool to induce an anti-NB immune response.

Based on these observations, we aimed to enhance the reported effect of the TH-directed DNA vaccine through co-expression of the immune stimulating cytokine interleukin 15 (IL-15) in the present study. IL-15 supports T cell proliferation and induction of long-lasting antigen-experienced CD8^+^ memory T cells [27–29]. In contrast to IL-2, which clearly has some clinical utility, IL-15 does not induce regulatory T cells (T_reg_) [30] and thus represents a promising alternative for therapeutic use. Here, we generated and characterized a new bicistronic xenogeneic DNA vaccine encoding both the TAA TH and the immune stimulating IL-15, and investigated *in vivo* effectivity against NB in mice after oral immunization.

## Results

### TH expression by human NB cells

**Fig 1:**
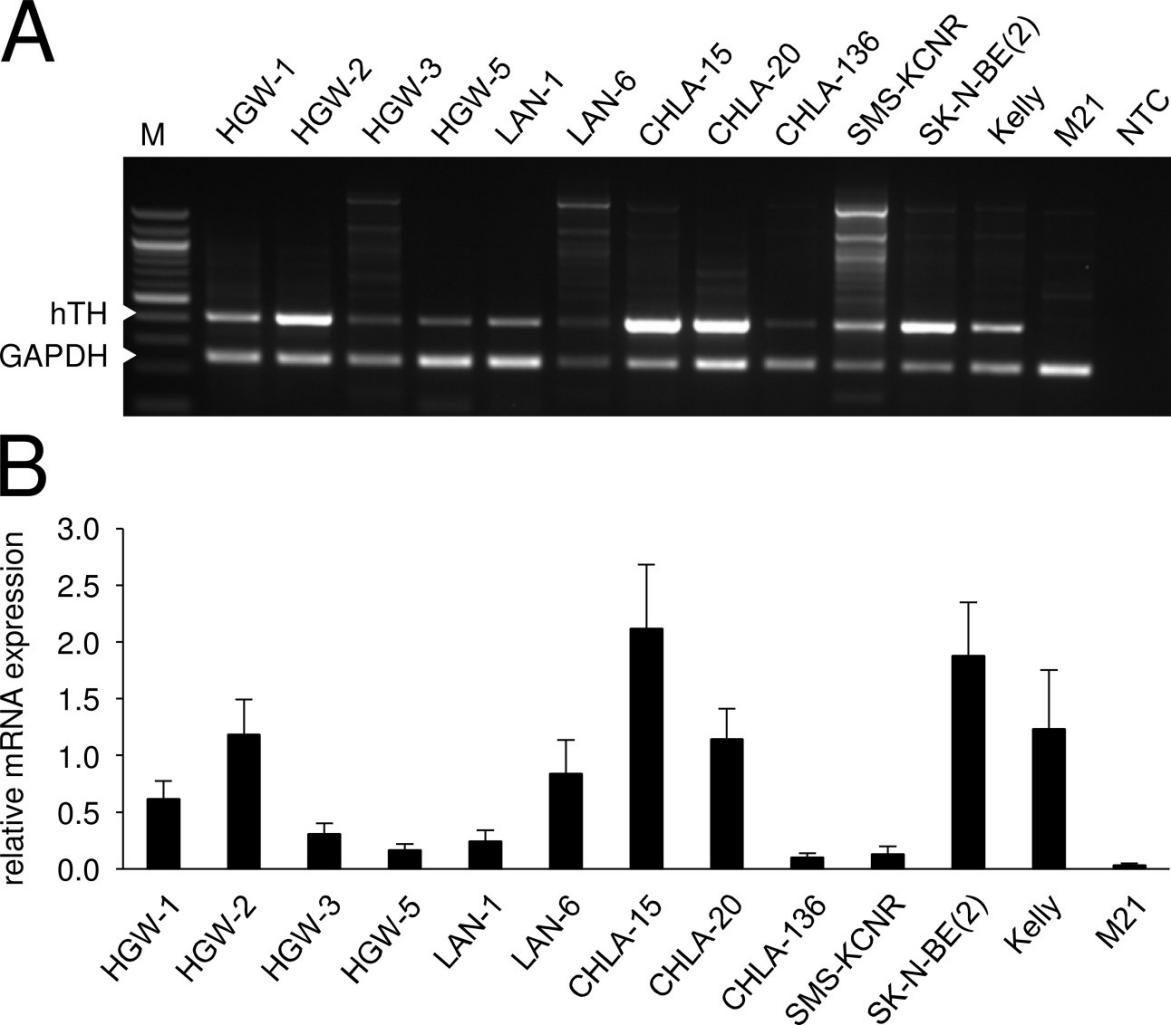
Analysis of hTH mRNA expression by NB cells. Representative RT-PCR (A) and densitometry analysis (B) of human TH (hTH) mRNA expression by human NB cell lines LAN-1, LAN-6, CHLA-15, CHLA-20, CHLA-136, SMS-KCN, SK-N-BE(2) and Kelly as well as by primary cell lines HGW-1, HGW-2, HGW-3, and HGW-5. hTH mRNA expression (A, upper panel, 405 bp) was quantified relative to the internal control GAPDH (A, lower panel, 238 bp) using densitometry according to the formula: hTH signal intensity/GAPDH signal intensity. Values are given as means ± SEM, n = 3. M: marker (100 bp, 0.1–1.5 kbp), NTC: no template control.

Reverse transcriptase-PCR (RT-PCR) analysis (Fig 1A) revealed different levels of human TH (hTH) mRNA expression (405 bp) by all tested human NB cells. Densitometry analysis (Fig 1B) in relation to glyceraldehyde 3-phosphate dehydrogenase (GAPDH; 238 bp) revealed higher levels of TH mRNA expression by HGW-1, HGW-2, LAN-6, CHLA-15, CHLA-20, SK-N-BE(2) and Kelly compared to HGW-3, HGW-5, LAN-1, CHLA-136 and SMS-KCN. These data emphasize the potential for this TAA as a target for vaccination against many NBs.

### Generation of the bicistronic TH-based DNA vaccine

**Fig 2:**
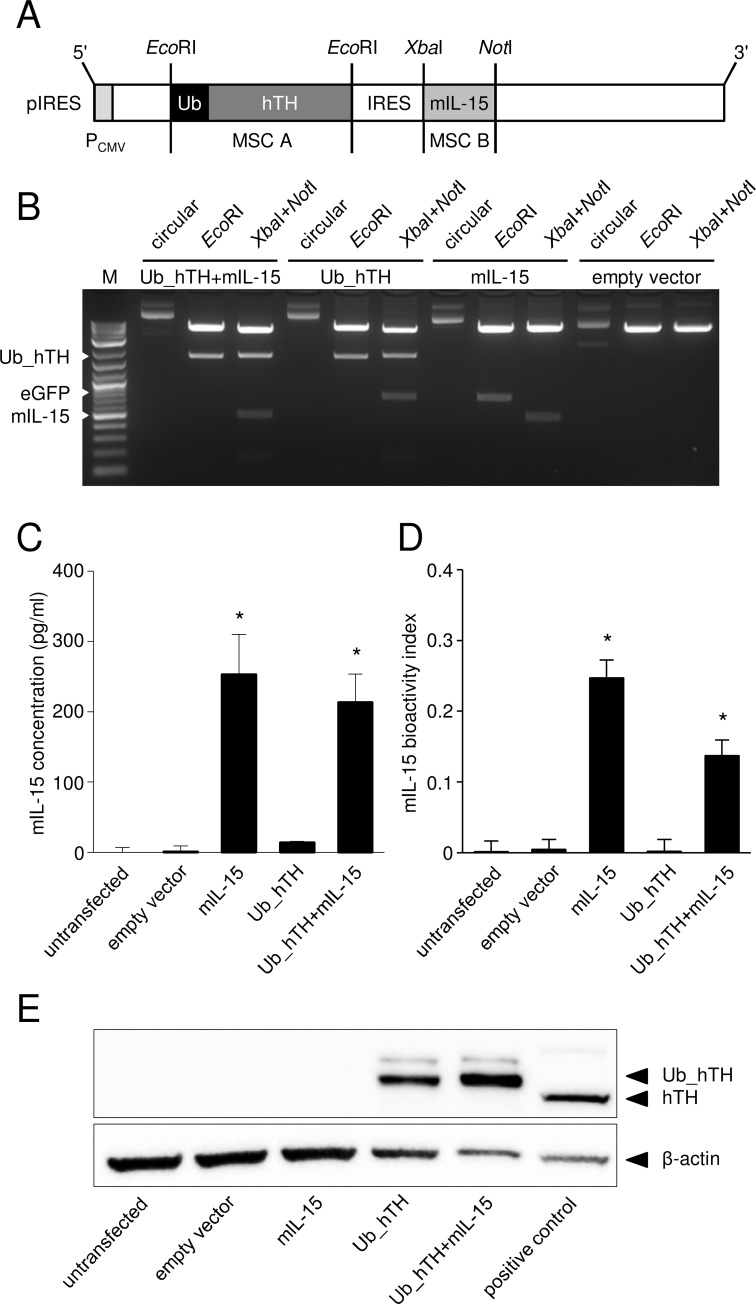
**Vaccine generation (A-B) and evaluation of vaccine-mediated IL-15-, and TH protein expression (C-E).** For vaccine generation, the human TH DNA sequence (hTH) combined with an upstream Ub sequence and the DNA sequence encoding murine IL-15 (mIL-15) were cloned into the corresponding MCS (A). eGFP was integrated into control plasmids. After stepwise ligation of the DNA fragments (Ub_hTH (1743 bp), mIL-15 (496 bp), eGFP (726 bp)), correct insertion was confirmed by restriction anaylsis using restriction enzymes *EcoR*I and *Xba*I/ *Not*I for MCS A and B, respectively(B). M: Marker (2-log, 0.1–10.0 kbp), P_CMV_: cytomegalovirus promoter. For determination of IL-15 secretion (C) and bioactivity (D), supernatants of CHO cells 30 h after transfection with IL-15 containing plasmids were analyzed by ELISA and a bioactivity XTT assay, respectively. mIL-15 bioactivity index was calculated as follows: OD_475nm_(supernatant sample)–OD_475nm_(media blank)–OD_660nm_(supernatant sample). Data are presented as mean ± SEM (n = 3). *P < 0.05 vs. empty vector control. For Ub_hTH protein (65 kDa) expression analysis (E), lysates of CHO cells transfected in the presence of lactacystin, were analyzed using SDS-PAGE and western blot technique. Lysates of murine adrenal gland served as positive control (mTH: 56 kDa) and β-actin as internal loading control.

After ligation of the DNA fragments Ub_hTH, mIL-15 and enhanced green fluorescent protein (eGFP), which served as control, into the bicistronic plasmid (Fig 2A), their correct insertion was confirmed by restriction analysis (Fig 2B). Gel electrophoresis of DNA fragments after digestion with *Eco*RI (multiple cloning site (MCS) A) or *Xba*I and *Not*I (MCS B) revealed the correct insertion of Ub_hTH (1743 bp), mIL-15 (496 bp) and eGFP (726 bp) into their respective MCSs. A final sequence analysis (LGC Genomics) confirmed the correct orientation and DNA sequence of the integrated fragments (data not shown).

### *In vitro* characterization of the bicistronic TH/IL-15-based DNA vaccine

#### Secretion and bioactivity of mIL-15

Analysis of IL-15 clearly showed significant increased release of 213±41 pg/ml and 253±57 pg/ml of IL-15 in respective supernatants from CHO cells transfected with the bicistronic TH/IL-15-based plasmid and the IL-15 control plasmid by ELISA (Fig 2C). Bioactivity of the secreted IL-15 was assessed with a CTLL-2 cell proliferation assay (Fig 2D) and a bioactivity index (BI) was calculated. Compared to controls, (BI for all: 0.0), we observed clear proliferation of CTLL-2 cells incubated with supernatants from CHO cells transfected with the bicistronic TH/IL-15-based- (BI: 0.14) or IL-15 control plasmid (BI: 0.25). These data clearly demonstrate the correct transcription and translation of DNA vaccine-encoded mIL-15 and adequate secretion of the bioactive cytokine by cells harboring the bicistronic plasmid.

#### Protein expression of hTH

The DNA vaccine-mediated expression of the TH protein (56 kDa) linked to Ub (9 kDa) was shown by western blot analysis (Fig 2E). TH_Ub (65 kDa) was detected in CHO cells transfected with the bicistronic TH/IL-15-based (lane 5) and TH control plasmid (lane 4). As expected, we observed TH expression (56 kDa; lane 6) in lysates of murine adrenal gland, used as positive control. These observations clearly demonstrate DNA vaccine-mediated synthesis of the hTH protein conjugated to Ub.

### NB-specific characterization of murine NXS2-HGW cells

**Fig 3:**
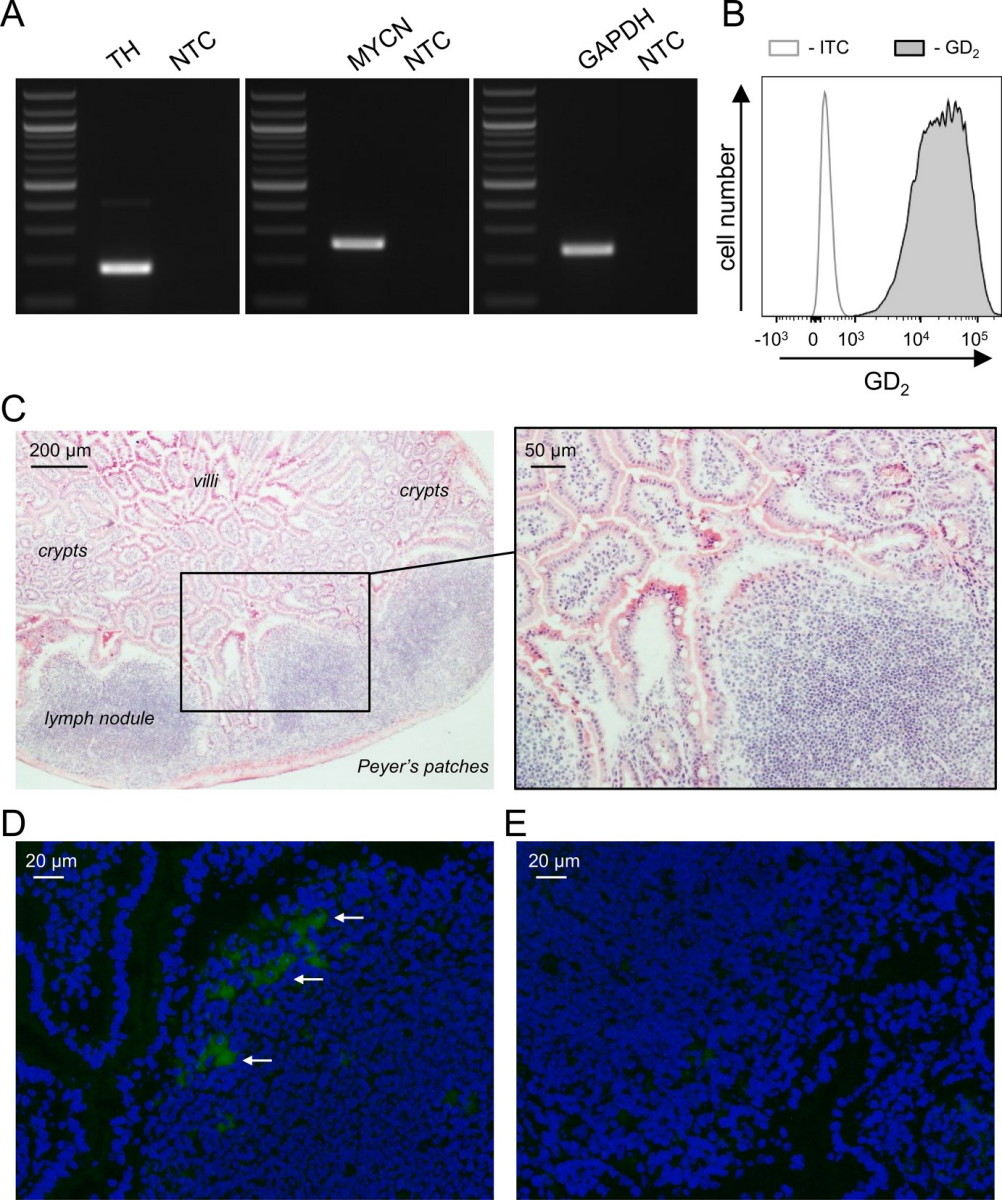
**Characterization of the murine neuroblastoma cells NXS2-HGW (A-B) and evaluation of DNA vaccine-induced gene delivery *in vivo* (C-D).** RT-PCR analysis of mRNA expression (TH, MYCN and GAPDH) by the tumor cells NXS2-HGW that were used for evaluation of anti-NB effects of the generated vaccine *in vivo* (A). NTC: no template control. For flow cytometry analysis of GD_2_ expression (B), NXS2-HGW cells were stained with Alexa 647 labeled anti-GD_2_ Ab (filled black curve) or respective isotype control (open gray curve). C-E: Mice (n = 3) were subjected to 4 oral vaccinations at seven day intervals with 5×10^7^ *salmonella typhimurium* (strain SL7207) carrying a plasmid encoding eGFP. For isolation of Peyer’s Patches, mice were sacrificed 72 h after last immunization. Cryo-sections (6 μm) were fixed followed by H&E staining (C; 40× and 100× magnification) or staining of the nucleus with DAPI for fluorescence microscopy (D; eGFP in green, DAPI in blue, 200× magnification). Arrows indicate area of green fluorescence representing plasmid incorporation. Peyer’s patches of mice inoculated with empty vector-bearing SL7202 served as negative control (E).

Prior to injection into mice, NXS2-HGW tumor cells were analyzed for NB-specific characteristics[31]. Gene-specific RT-PCR analysis revealed a strong mRNA expression of the TAA TH (175 bp; Fig 3A), underlining the applicability of this model to investigate the TH-directed immunotherapy. We also observed a strong expression of two other NB-specific markers MYCN (248 bp, Fig 3A) and GD_2_ (Fig 3B) using RT-PCR- and flow cytometry analysis.

### Evaluation of DNA vaccine-mediated anti-NB effect *in vivo*

#### DNA vaccine-induced gene delivery

Immunization of mice using the pIRES plasmid encoding the reporter gene eGFP resulted in clear eGFP expression (green fluorescence) in antigen presenting cells of the Peyer’s patches compared to negative control (Fig 3C–3E). These observations indicate an effective gene transfer *in vivo* after oral DNA vaccination using the attenuated *salmonella typhimurium* strain SL7202.

#### Effects of vaccination on tumor growth

**Fig 4:**
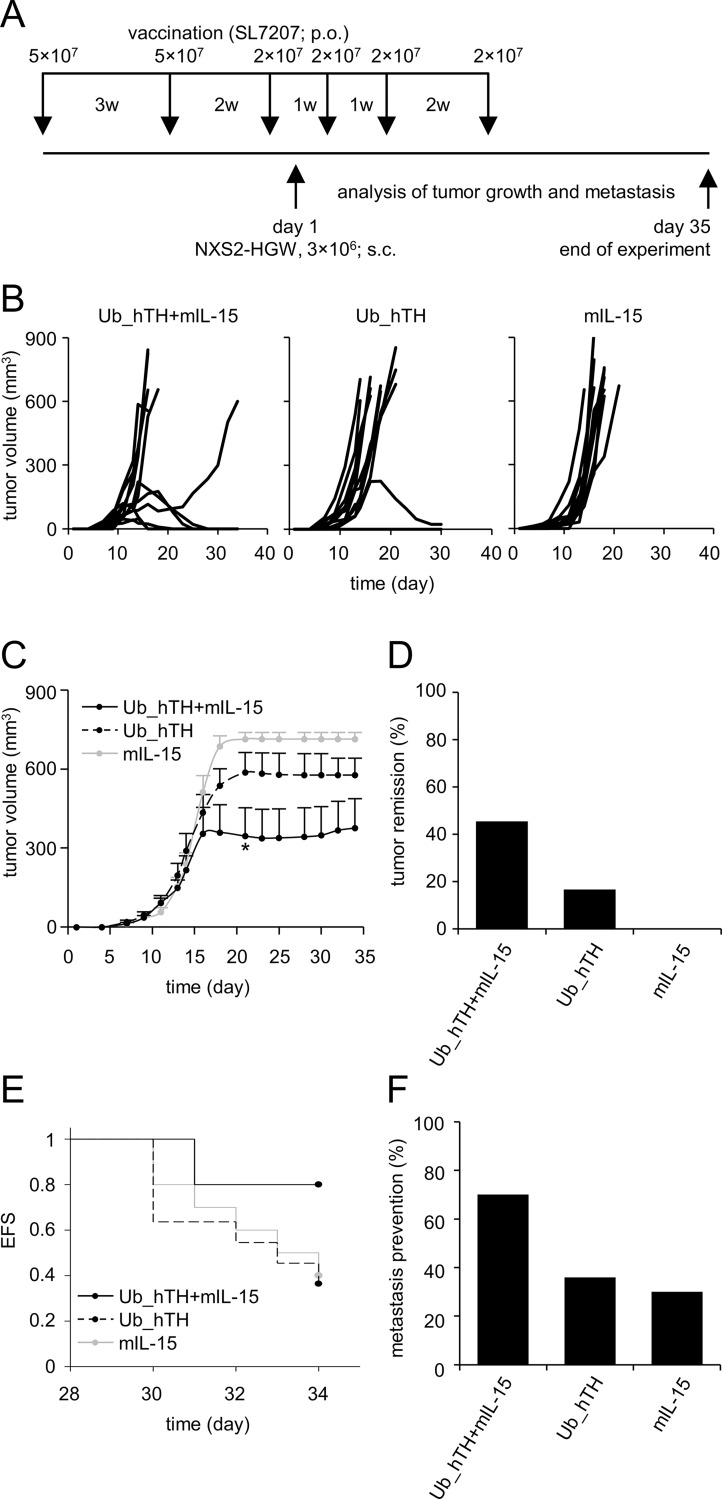
*In vivo* evaluation of anti-tumor effects of the bicistronic TH/IL-15-based DNA vaccine. Schematic overview of the *in vivo* experiment (A). A/J mice (n = 12 per group) were vaccinated with plasmid bearing attenuated *S*. *typhimurium* (SL7207; p.o.) 3× prior and 3× after s.c. injection of 3×10^6^ NB cells NXS2-HGW. w: week. Mice were examined daily for determination of primary tumor growth and occurrence of metastasis until day 35. Primary tumors were surgically removed when their size exceeded 650 mm^3^. Tumors were measured using a caliper followed by tumor volume calculation according to the formula: (length×width×height)/2. Tumor growth of individual mice (B) and average tumor volume per group (C) of mice treated with the bicistronic DNA vaccine (Ub_hTH+mIL-15, solid black line) or the control plasmids (Ub_hTH, dashed black line and mIL-15, grey line) are shown. Tumor volume of individual mice at the time point of tumor resection was included in calculation of the average tumor volume on subsequent time points. Values for tumor volume are given as means ± SEM (n = 12) in cubic millimeters. *P<0.05 vs. mIL-15. Proportion comparison of mice showing tumor remission (D). Evaluation of event-free survival (EFS) regarding occurrence of palpable metastasis that is defined as event between day 28 and 34 (E, *P* = 0.125). After tumor resection, mice were examined and spontaneous metastasis was determined from day 28 to day 34 by daily palpation. Prevention of metastasis at the end of the experiment, assessed trough post-mortem examination (lymph node, ovary, liver, adrenal gland and abdomen), is given as proportion of mice without metastasis (day 35, F).

All mice vaccinated (vaccination schedule in Fig 4A) with the IL-15 control plasmid exhibited tumor progression (right panel, Fig 4B). The TH-based immunization resulted in improved outcome showing tumor remission for 16.7% of mice (2/12 mid panel, Fig 4B and 4D). The bicistronic TH/IL-15-based vaccine was the most effective against NB exhibiting tumor remission in 45.5% of mice (5/11 left panel, Fig 4B and 4D). These results were confirmed by calculating the group-specific average of tumor volume at each day of analysis. Here, our data demonstrate a slight reduction of tumor growth after vaccination with the plasmid encoding TH compared to the IL-15 control plasmid. Importantly, the application of the bicistronic TH/IL-15-based DNA vaccine significantly reduced tumor volume (Ub_hTH+mIL-15 vs. mIL-15, day 21, *P*<0.05; Fig 4C). These results demonstrate that IL-15 co-expression enhances anti-tumor effectivity of the TH-based DNA vaccination.

#### Effects of vaccination on spontaneous metastasis

To additionally evaluate the immune response concerning spontaneous metastasis, mice were examined for occurrence of metastasis during (day 28 to 34, daily palpation) and at the end of the experiment (day 35, post-mortem examination). From day 28 to day 34, the application of the bicistronic TH/IL-15-based DNA vaccine induced a trend towards reduction in the development of spontaneous metastasis (data not shown) and consequently improved EFS *P* = 0.125; Fig 4E) compared to the Ub_hTH- or mIL-15 plasmid, showing the impact of IL-15 co-expression on the induction of a long-lasting immunity. These data could be confirmed by the analysis at day 35 demonstrating that 70% of mice immunized with the bicistronic DNA vaccine were free of spontaneous metastasis (data on assessment of metastasis in lymph node, ovary, liver, adrenal gland and abdomen of individual mice not shown). In contrast, only 36% and 30% of mice receiving the plasmids encoding either Ub_hTH or mIL-15, respectively, were free of metastasis (Fig 4F). These results indicate a robust immune response against NB mediated by the TH/IL-15-based DNA vaccine and may point toward the induction of a persistent immunity.

#### Effects of vaccination on tumor infiltrating CTL and T_reg_

**Fig 5:**
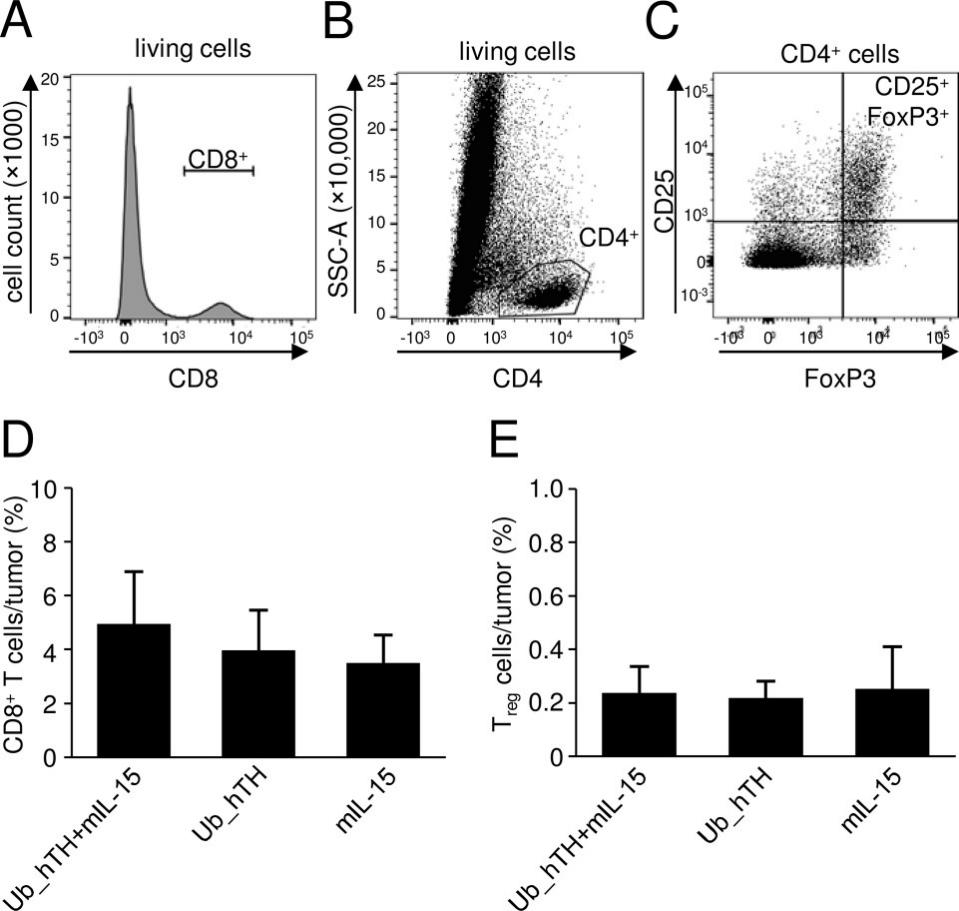
Effects of vaccination on tumor infiltrating CTL and T_reg_. Infiltration of tumor tissue by CD8^+^ and T_reg_ cells was analyzed by flow cytometry. Representative histogram (A; CD8^+^) and dot plots (B and C; gating for T_reg_) are shown. CD8+ and CD4+ fraction and CD25^+^FoxP3^+^ fraction were gated on lining cells and CD4+ cells, respectively. After preparation of a single cell suspension from resected tumors, cells were stained either with an anti-CD8 (A and D) or T_reg_ antibody pannel (CD4^+^, CD25^+^, FoxP3^+^; B, C and E). Cell subsets were calculated as a percent of positive cells (CD8^+^ or CD4^+^CD25^+^FoxP3^+^) within the living cell fraction. Values given are means ± SEM, n = 4.

We analyzed frequencies of tumor infiltration by CD8^+^ T cell and T_reg_ populations using flow cytometry (Fig 5A–5C). Surprisingly, in contrast to our results showing the reduction of tumor growth and prevention of metastasis, we did not detect any significant change of CD8^+^ T cell proportion among all groups (Fig 5D). This result suggests a highly immune suppressive tumor milieu at time point of tumor analysis.

Quantification of intratumoral T_reg_ did not show any differences among all experimental groups (Fig 5E), consistent with an immune stimulating role of IL-15 in the induction of an active immune response after DNA vaccination without induction of T_reg_.

## Discussion

The application of DNA-based cancer vaccines encoding TAAs offers a promising treatment strategy resulting in induction of a robust Th1 immune response [32]. However, many DNA vaccines successfully evaluated in animal models induced only moderate immune responses in humans [33–36]. This can be partially explained by the species-specific differences in toll-like receptor 9 expression that activates the immune response to bacterial and viral DNA [37].

In this study, the DNA vaccine was administered using live-attenuated *S*. *typhimurium*, that has multiple ligands for a broad range of toll-like receptors (e.g. bacterial wall-derived LPS for toll-like receptor 4 pathway stimulation facilitating antigen presentation), and is an established vaccine vehicle to optimize both humoral and cellular immune responses[15, 38]. To increase immunogenicity of DNA vaccination, we used xenogeneic DNA, encoding highly homologous proteins that are foreign to the species to be vaccinated. Additionally, we aimed to enhance anti-tumor responses by co-administration of the immune stimulatory cytokines IL-15.

Immunological approaches such as active immunization using a DNA vaccine have become more attractive for cancer patients, especially due to the development of a life-long protection for refractory patients [39, 40]. One clinical marker of NB patients is TH, the first-step enzyme of catecholamine biosynthesis [23, 24], which is an attractive target for immunotherapie against NB. We confirmed the stable expression of TH in different well-characterized human NB cell lines as well as in primary cell lines derived from tumors of high-risk NB patients. In line with that, *Huebener et al*. showed high TH expression in human NB tumors independent of disease stage [21]. These results, together with the fact that vaccination with TH-based DNA vaccines induced efficient anti-NB immune responses in mice without causing autoimmunity [16, 21, 25, 26], strongly supports our aim to generate a TH-based DNA vaccine for active anti-NB immunotherapy.

In this study, we generated, characterized and showed *in vivo* effectivity of a new bicistronic TH-based xenogenic DNA vaccine additionally encoding the cytokine IL-15. We hypothesized, that IL-15 directly released into the antigen presentation microenvironment, would enhance CTL activation resulting in generation of TAA-specific memory T cells.

Since generation and survival of memory lymphocytes require not only antigenic stimulation but also production of co-stimulatory cytokines in the periphery [41], the co-expression of immune stimulatory cytokines using DNA vaccines is a favorable strategy that reduces the cytokine-related effects associated with system application.

Interestingly, the integration of IL-12 which is involved in differentiation and homeostasis of effector and memory CD8^+^ T cells [42] was not able to enhance the potency of the TH-directed vaccination as previously reported by our lab [21]. With significant impact on CD8^+^ T cell turnover, IL-15 appeared to be effective against tumors in the context of immune therapy and genetic vaccines [30, 43, 44] and showed preventive potential in human diseases, such as HIV infections [30, 41]. These findings strongly supported our idea to introduce IL-15 as a suitable adjuvant for TH-directed immunization. Indeed, our data clearly demonstrated biological activity of DNA-vaccine-derived mIL-15 as an immune stimulating cytokine. In contrast to other cytokines, biological activity of IL-15 is mainly mediated by *trans*-presentation of the cytokine to target cells by the cell-bound type-specific α-moiety of its receptor (IL-15Rα) which is constantly expressed by IL-15 producing cells, such as antigen presenting cells [28, 45–47]. In this context, it was reported that the IL-15/IL-15Rα complex exhibited hyperagonist activity toward CD8^+^ memory T cells [48–50] suggesting that the IL-15 mechanism of action *in vivo* could be augmented by the administration of receptor-complexed IL-15 [47]. Besides, another study showed that high dose of plasmid-encoded IL-15 could even inhibit the immune response of an influenza DNA vaccine [51] suggesting that high levels of IL-15 activity might not have the desired effect for different vaccination strategies. These results supported the use of this cytokine without its receptor in combination with our TH-directed DNA vaccine. Importantly, co-expression of IL-15 markedly enhanced the TH-specific anti-NB effect in our NB mouse model. We could show a strong reduction of tumor growth and spontaneous metastasis resulting in an increased tumor remission rate of mice vaccinated with the bicistronic plasmid compared to a TH-directed vaccination alone. Especially the reduction of metastasis may indicate the induction of a long-lasting immune protection which is generally mediated by memory T cells. This underlines the role of cytokines in induction of a Th1-directed immune response after DNA vaccination.

Furthermore, after immunization with the IL-15-containing bicistronic DNA vaccine, we did not observe an increase in tumor-infiltrating T_reg_, known as poor prognostic factor [52],. This is in line with *Berger et al*., who showed that IL-15 treatment did not expand the CD4^+^/CD25^+^/FoxP3^+^ T cell subset in peripheral blood [29].

Although, vaccination with the bicistronic TH/IL-15-based DNA vaccine resulted in enhanced tumor remission, we did not detect significantly increased levels of tumor-infiltrating CD8^+^ T cell. This is in contrast to the finding of *Huebener et al*. reporting a 5-fold increase of infiltrating CD8^+^ T cells into primary tumors after vaccination with a xenogenic TH-based DNA vaccine 15 days after tumor cell implantation (tumor volume of about 200–400 mm^3^). Differences in time point of tumor analysis, which is indeed a crucial factor for analysis of tumor-infiltrating effector cell frequencies, could explain this discrepancy. In contrast to *Huebener et al*., we analyzed tumors at a more advanced bulky state (650 mm^3^) and small tumors that went into tumor remission were excluded from CD8^+^ T cell analysis. In this context, it was reported that the proportion of tumor-infiltrating CD8^+^ CTLs was diminished during tumor growth [53], exhibiting a shift of the immune profile within the tumor microenvironment from Th1 to Th2. Th1-profiled immune cells present in the microenvironment during the early phase of tumor development promote immune surveillance whereas during the later phase a shift towards the Th2 immune response diminishes cytotoxic T cell responses resulting in tumor growth [53, 54]. Hence, analysis of tumor-infiltrating CD8^+^ T cells at an early tumor stage before the Th1/Th2 shift-induced redistribution of the T cell compartment occurs is probably more feasible to detect differences among experimental groups.

In summary, we generated and characterized a bicistronic xenogenic DNA vaccine encoding the NB-associated antigen TH and the immune stimulatory cytokine IL-15. The immune response induced by TH-directed vaccination was further enhanced by the co-expression of IL-15 resulting in protection of mice from tumor growth and prevention of spontaneous metastasis after a lethal challenge with NB. We demonstrated that DNA vaccine delivery by attenuated bacteria in combination with vaccine-mediated ubiquitination of the TAA resulted in successful gene transfer to the antigen presenting cells of Peyer’s patches providing a promising therapeutic strategy to induce effective antitumor immune response in NB.

## Materials and methods

### Cell lines and animal experiments

All cell lines were cultured at 37°C, 5% CO_2_ in media supplemented with heat inactivated 10% FCS (Capricorn Scientific GmbH), 100 U/ml penicillin and 0.1 mg/ml streptomycin (PAN BIOTECH). Murine NXS2-HGW[31, 55] and Chinese Hamster Ovary (CHO) cells were cultured in high-glucose DMEM (Capricorn Scientific GmbH). RPMI 1640 (Capricorn Scientific GmbH) was used for human LAN-1, LAN-6, SMS-KCN, SK-N-BE(2) and Kelly. For CTLL-2 culture, RPMI was supplemented with 100 U/ml IL-2 (Novartis). Human CHLA-15, CHLA-20, CHLA-136 and primary cell lines derived from tumors of high-risk NB patients (HGW-1, HGW-2, HGW-3 and HGW-5[56]) were cultured in IMDM with 20% FCS. *E*. *coli* and SL7207 *S*. *typhimurium* were cultured in LB-medium (Roth) containing 50 μg/ml ampicillin (AppliChem) (LB-amp-medium). Animal experiments were approved by the animal welfare committee (Landesamt für Landwirtschaft, Lebensmittelsicherheit und Fischerei Mecklenburg-Vorpommern, LALLF M-V/7221.3-1-082/16) and supervised by the commissioner for animal welfare at the University Medicine Greifswald representing the Institutional Animal Care and Use Committee (IACUC). Female A/J mice (8 weeks, Charles River Laboratories) were housed in standard animal laboratories (12 h light/dark cycle) with free access to water and standard laboratory chow *ad libitum*and immunized by oral gavage of SL7207 bacteria at indicated time points (Fig 4A). Tumor cells (3×10^6^) were injected subcutaneously into the left flank of A/J mice. Primary tumor volume was analyzed using micro caliper measurements and calculated according to the formula: (length×width×height)/2. When primary tumors reached a volume of 650 mm^3^ and/or showed signs of necrosis, they were surgically removed and cryoconserved in O.C.T. compound mounting medium (VWR) for further analysis. Tumor volume of individual mice at the time point of tumor resection was included in calculation of the average tumor volume on subsequent time points. After tumor resection, spontaneous metastasis (lymph node, ovary, liver, adrenal gland and abdomen) was determined from d28 to d34 and at the end of the experiment (d35) by daily palpation and post-mortem examination, respectively.

### Analysis of TH-, MYCN- and GD_2_ expression by NB cells

Expression of TH and MYCN mRNA by human NB cells and the murine NB cells NXS2-HGW was analyzed by RT-PCR (Table 1). GAPDH served as endogenous control. First, total RNA was isolated from 2×10^6^ cells using the Nucleo Spin RNA II Kit (Macherey Nagel) according to the manufacturer’s guidelines. Next, 2 μg of total RNA were used for cDNA synthesis with qScript cDNA Synthesis Kit (Quanta Biosciences) followed by gene-specific PCR analysis (Table 1). Expression of GD_2_ was analyzed using flow cytometry. Therefore, 1×10^6^ NXS2-HGW cells were stained with Alexa647-labeled mouse anti-GD_2_ Ab (0.63 μg/ml, clone 14G2a) in staining buffer (1% BSA, 0.1% EDTA, and 0.1% NaN_3_ in 1×PBS). Alexa647-labeled mouse IgG2a Ab (MOPC-173, Biolegend) served as isotype control (ITC).

**Table 1:** Primer sequences and PCR conditions.

Gene	Primer sequence (5′ - 3′)	PCR condition	number of cycles	product size (bp)
*mTH*	f: GCCGTCTCAGAGCAGGATAC	D: 95°C, 15 s	25	175
	r: CGAATACCACAGCCTCCAAT	A: 56°C, 15 s		
		E: 72°C, 15 s		
*hTH*	f:GGCCCAAGGTCCCCTGGTTC	D: 95°C, 30 s	27	405
	r: ACAGCAGGCCGGCCACAGGC	A: 62°C, 30 s		
		E: 72°C, 30 s		
*mMYCN*	f: GCTGCGGTCACTAGTGTGTC	D: 95°C, 15 s	28	248
	r: CGCACAGTGATCGTGAAAGT	A: 55°C, 15 s		
		E: 72°C, 30 s		
*mGAPDH*	f: AACTTTGGCATTGTGGAAGG	D: 95°C, 15 s	23	223
	r: ACACATTGGGGGTAGGAACA	A: 56°C, 15 s		
		E: 72°C, 15 s		
*hGAPDH*	f: GAGTCAACGGATTTGGTCGT	D: 95°C, 30 s	27	238
	r: TTGATTTTGGAGGGATCTCG	A: 62°C, 30 s		
		E: 72°C, 30 s		
*Ub_hTH*	f: GAATTCATGCAGATTTTCGTGAAGACCCT	D: 95°C, 15 s	27	1749
(MCS A)	r: GAATTCCTAGCCAATGGCACTCAGCGCAT	A: 70°C, 15 s		
		E: 72°C, 110 s		
*mIL-15*	f: TCTAGAATGAAAATTTTGAAACCATATA	D: 95°C, 15 s	30	503
(MCS B)	r: GCGGCCGCTCAGGACGTGTTGATGAACAT	A: 63°C, 15 s		
		E: 72°C, 30 s		
*eGFP*	f: GAATTCATGGTGAGCAAGGGCGAGG	D: 95°C, 15 s	24	732
(MCS A)	r: GAATTCTTACTTGTACAGCTCGTCC	A: 71°C, 30 s		
		E: 72°C, 45 s		
*eGFP*	f: TCTAGAATGGTGAGCAAGGGCGAGG	D: 95°C, 15 s	24	734
(MCS B)	r: GCGGCCGCTTACTTGTACAGCTCGTCC	A: 71°C, 30 s		
		E: 72°C, 45 s		

Gene-specific primer sequences for murine and human tyrosine hydroxylase (mTH and hTH) and murine MYCN (mMYCN) were used for analysis of mRNA expession. Murine and human GAPDH (mGAPDH and hGAPDH) served as internal controls. Amplification of Ub_hTH, mIL-15 or eGFP was accomplished by PCR using specific primers that included restriction sites (underlined) suitable for integration into respective MSCs of the bicistronic plasmid pIRES. PCR reactions consisted of an initial denaturation phase at 95°C for 2 min followed by a number of gene-specific cycles, each comprising the three phases: denaturation (D), primer annealing (A) and elongation (E). Each PCR reaction was completed by a final elongation phase at 72°C for 2 min. f: forward primer, r: reverse primer.

### Generation of the bicistronic TH/IL-15-based DNA vaccine

The mammalian bicistronic expression vector pIRES (Clontech) allowing co-transcription of two genes was used for vaccine generation. First, the cDNA sequences encoding hTH [21] linked to Ub (A_76_) and mIL-15 were amplified by PCR using primers (Table 1) that include restriction sites of the respective MCS for ligation into pIRES. Initially, both amplicons were ligated into the pCR2.1-TOPO TA cloning vector (Invitrogen) followed by transformation of *E*. *coli*. After plasmid DNA isolation using NucleoSpin Plasmid Kit (Macherey-Nagel) and sequence analysis (LGC Genomics), both inserts were digested by respective restriction enzymes (*Eco*RI for MCS A and *Xba*I and *Not*I for MCS B) and subcloned into the pIRES vector (Fig 2A). For further *in vitro* and *in vivo* analysis, the bicistronic DNA plasmid was propagated using *E*. *coli*. For generation of control plasmids containing either the sequences of Ub_hTH or mIL-15, the coding sequence of eGFP was additionally ligated into the respective free MCS.

### *In vitro* characterization of the bicistronic TH/IL-15-based DNA vaccine

#### Analysis of mIL-15 secretion and bioactivity

In order to confirm synthesis and successful secretion of IL-15, CHO cells were transiently transfected with the IL-15-containing plasmids using jetPRIME transfection reagent (Polyplus-transfection) according to the manufacturer's protocol. After 30 h, supernatants were collected and analyzed by the mouse IL-15 DuoSet ELISA (R&D Systems, bio-techne) according to the manufacturer's instructions. The bioactivity of mIL-15 secreted from transfected CHO cells was determined using a XTT proliferation assay [57]. Prior to analysis, IL-15-sensitive CTLL-2 cells were cultured for 24 h using cytokine-free medium. Next, cells were harvested, seeded into a 96-well plate (7.5×10^4^ cells/well) and cultivated for 24 h with the supernatants of transfected CHO cells in triplicates. Proliferation was analyzed using a standardized colorimetric XTT-Assay.

#### Analysis of hTH protein expression

The TH protein expression was determined in CHO cells 30 h after transient transfection with the TH containing plasmids in combination with the proteasome inhibitor Lactacystin (10 μM, Enzo) to inhibit Ub-mediated proteasomal degradation of TH. Cells were lysed (lysis buffer containing 100 mmol/l KCl, 20 mmol/l NaCl, 2 mmol/l MgCl_2_, 0.96 mmol/l NaH_2_PO_4_, 0.84 mmol/l CaCl_2_, 1 mmol/l EDTA, 25 mmol/l HEPES, pH 7.2 and protease inhibitor cocktail (SigmaAldrich), 30 sec ultrasound), centrifuged (10,000 g, 4°C, 10 min) and 30 μg total protein was used for SDS-polyacrylamide gel electrophoresis (SDS-PAGE, BioRad). After proteins were transferred onto a nitrocellulose membrane (iBlot, Invitrogen), blocking buffer (5% BSA, 0.1% Tween 20 in 1×TBS) was added for 1 h at room temperature, followed by incubation with anti-TH Ab (polyclonal human and mouse specific, diluted 1:1,000 in blocking buffer; CellSignaling) at 4°C, overnight and washed three times in 1×TTBS (0.1% Tween 20 in 1×TBS). Subsequently, peroxidase-labeled anti-rabbit IgG Ab (CellSignaling) was added (1:2,000 in blocking buffer). The membrane was washed three times (1×TTBS, 5 min, room temperature) and signals were analyzed with the ECL Detection Reagents (BioRad). Blots were analyzed using ImageLab 3.0 software (BioRad). Lysate of murine adrenal gland served as positive control.

### Transformation of attenuated *S*. *typhimurium* with the bicistronic DNA vaccine

The attenuated *S*. *typhimurium* strain SL7207 (AroA^-^) was used as vaccine vehicle for oral immunization of mice as described previously [21]. In brief, prior to transformation, bacteria were cultivated and harvested in a mid-log growth phase. After two wash steps (ice-cold water, 4,000×g, 10 min, +4°C), 1×10^9^ SL7207 were electroporated with 40 ng purified plasmid DNA (2.5 kV, 600 Ω, 10 μF, 5 ms) followed by seeding onto LB-amp-agar plates. A single SL7207 clone was then expanded in LB-amp-medium, harvested during a mid-log growth phase, washed with ice-cold water and resuspended in tap water for immunization (concentrations indicated in Fig 4A).

### Evaluation of DNA vaccine-induced gene delivery *in vivo*

For evaluation of general DNA vaccine-mediated gene delivery to antigen presenting cells of the Peyer’s Patches, mice (n = 3) were vaccinated with 5×10^7^ SL7202 transformed with the eGFP plasmid four times in a seven-day interval. 72 h after the last immunization, cryo-sections (6 μm) containing Peyer’s Patches were prepared and fixed with 4.5% formaldehyde for 20 min at room temperature followed by staining of the nuclei with 4',6-diamidino-2-phenylindole (DAPI) (5 min, room temperature, 0.8 μg/ ml in 1×PBS). After mounting (Mowiol medium), images were captured on a fluorescence microscope (Olympus) for visualization of DAPI (excitation 325–375 nm, emission 435–485 nm) and eGFP (excitation 450–490 nm, emission 500–550 nm) fluorescence. To show a compartment overview of the isolated Peyer’s Patches, cryo-sections were stained with Mayer hematoxylin solution for 4 min followed by washing in warm running tap water for 10 minutes. After a short rinse in distilled water, sections were counterstained in eosin Y solution for 4 min, dehydrated using an ascending alcohol series (70%, 96%, 99.8%) and mounted using Roti-Histokitt II (H&E stain).

### Analysis of tumor-infiltrating T_reg_ and CD8^+^ effector cells

For analysis of tumor-infiltrating T_reg_ (CD4^+^/CD25^+^/FoxP3^+^) and CTLs (CD8^+^) using flow cytometry, a single cell suspension was prepared from resected tumors using a cell strainer (70 μm). For T_reg_ analysis, viability staining based on the fixable viability dye eFluor 506 (diluted 1:1,000 in PBS, eBioscience) was performed. Prior to the incubation with FITC-labeled anti-mouse CD4 Ab (2.5 μg/ml, clone GK1.5, Biolegend) and PE/Cyanine7 (Cy7)-labeled anti-mouse CD25 Ab (1.25 μg/ml, clone PC61.5, eBiosciences) in 100 μl staining buffer (2% FCS, 1 mM EDTA in 1×PBS), nonspecific binding sites were saturated using mouse FcR Blocking Reagent (Miltenyi Biotec) according to the manufacture’s protocol. FITC-labeled rat IgG2b Ab (clone RTK4530, Biolegend) and PE/Cy7-labeled rat IgG1 Ab (clone eBRG1, eBiosciences) were used as ITC, respectively. Next, cells were fixed, permeabilized, and intracellularly stained with APC-labeled anti-mouse FoxP3 Ab (0.44 μg/ml, clone 3G3, Miltenyi) or APC-labeled mouse IgG1 Ab (clone MOPC-21, Biolegend) as ITC using the FoxP3 Staining Buffer Set (Miltenyi). CTLs were stained with PerCP-labeled anti-mouse CD8a Ab (2 μg/ml, clone 53–6.7, BD) in 100 μl staining buffer (1% BSA, 0.1% EDTA, and 0.1% NaN3 in 1×PBS). PerCP-labeled rat IgG2a,κ Ab (clone R35-95, Biolegend) served as ITC. Cells were examined using the BD flowcytometer FACSCANTO II and FACS Diva software (BD Biosciences). Data were analyzed with FlowJo 10.4 software (Treestar). Frequencies of T_reg_ (CD4^+^, CD25^+^, FoxP3^+^) and CTL (CD8^+^) cell subsets were calculated as a percent of positive cells within the live cell fraction (Fig 5A–5C).

### Statistical analysis

Statistical analysis was performed using Sigma Plot software (Jandel Scientific Software). Differences between groups were assessed using non-parametric Mann-Whitney U test. For more than two groups, the non-parametric Kruskal-Wallis test was used followed by appropriate post hoc comparison. A *P* value of < 0.05 was considered as significant. All data are presented as mean ± SEM (standard error of the mean). Survival probabilities were estimated using Kaplan-Meier analysis and compared using LogRank statistics (Mantel-Cox). For event-free survival, first notable metastasis detected by palpation was defined as event.
